# Impact of Accurate Detection of Freeway Traffic Conditions on the Dynamic Pricing: A Case Study of I-95 Express Lanes

**DOI:** 10.3390/s21185997

**Published:** 2021-09-07

**Authors:** Suhaib Alshayeb, Aleksandar Stevanovic, Nikola Mitrovic, Branislav Dimitrijevic

**Affiliations:** 1 Department of Civil & Environmental Engineering, Swanson School of Engineering, University of Pittsburgh, Benedum Hall, 3700 O’Hara Street Pittsburgh, Pittsburgh, PA 15261, USA; stevanovic@pitt.edu; 2 A&P Consulting Transportation Engineers, 8935 NW 35th Ln, Doral, FL 33172, USA; nmitrovic@apcte.com; 3 John A. Reif, Jr. Department of Civil and Environmental Engineering, New Jersey Institute of Technology, University Heights, Newark, NJ 07102, USA; dimitrijevic@njit.edu

**Keywords:** express lanes, dynamic toll, accurate detection, speed-volume relationship, HOT lanes, congestion pricing

## Abstract

Express lanes (ELs) implementation is a proven strategy to deal with freeway traffic congestion. Dynamic toll pricing schemes effectively achieve reliable travel time on ELs. The primary inputs for the typical dynamic pricing algorithms are vehicular volumes and speeds derived from the data collected by sensors installed along the ELs. Thus, the operation of dynamic pricing critically depends on the accuracy of data collected by such traffic sensors. However, no previous research has been conducted to explicitly investigate the impact of sensor failures and erroneous sensors’ data on toll computations. This research fills this gap by examining the effects of sensor failure and faulty detection scenarios on ELs tolls calculated by a dynamic pricing algorithm. The paper’s methodology relies on applying the dynamic toll pricing algorithm implemented in the field and utilizing the fundamental speed-volume relationship to ‘simulate’ the sensors’ reported data. We implemented the methodology in a case study of ELs on Interstate-95 in Southeast Florida. The results have shown that the tolls increase when sensors erroneously report higher than actual traffic demand. Moreover, it has been found that the accuracy of individual sensors and the number of sensors utilized to estimate traffic conditions are critical for accurate toll calculations.

## 1. Introduction

The last few decades have seen a constant increase in traffic congestion in urban areas, primarily driven by the growth in travel demand. Traffic congestion has numerous negative impacts on society, including unnecessary delays, increased commuting times, excessive fuel consumption, air pollution, and other externalities [1]. Depending on the context and causes of traffic congestion, various strategies have been developed and implemented to mitigate its adverse effects. One strategy for dealing with traffic congestion on freeways is to apply road congestion pricing [2]. One of the applications of road congestion pricing is the implementation of high occupancy toll (HOT) and express-toll lanes. Such lanes are generally implemented to achieve an improved operational condition on a highway.

The express-toll lanes (ELs) have emerged from the concept of high-occupancy vehicle lane (HOV) systems to increase the use of available roadway capacity. ELs are a type of managed lanes where most users are subject to a toll, while some users may be exempted from paying the toll. For example, Interstate-95 ELs could be used by registered carpools (3+ occupants, hybrids, and South Florida Vanpools) at no charge [3]. Unlike toll roads, drivers have a choice not to pay the EL toll and use general-purpose (GP) lanes.

Generally, ELs systems can support up to five primary pricing or ‘toll’ modes: (i) closed operations, usually applied only during maintenance or in response to traffic incidents; (ii) zero toll, implemented in support of evacuations (e.g., during hurricanes); (iii) manual toll, which allows setting a fixed toll rate; (iv) day-of-week/time-of-day toll, which implements preconfigured toll rates based on the day of the week and the time of day; (v) dynamic toll, where tolls are adjusted dynamically over time, based on a pre-defined algorithm that accounts for roadway traffic demand [3].

In the past few years, dynamic toll pricing has gained popularity (over fixed tolls and time-of-day toll pricing) as a more effective strategy for controlling traffic jams [4]. Since the dynamic pricing algorithms use real-time traffic data such as speeds and volumes, the reliability of toll calculations heavily depends on the number and accuracy of the volume and speed sensors. Inaccurate or missing data reported by sensors can result in toll overcharging, which might repel motorists from using the ELs facility, or toll undercharging, which may cause a loss of revenue for the operating agencies and reduce the effectiveness of the ELs facility. That suggests that the detection technology implemented for dynamic tolling should satisfy a certain level of accuracy and store traffic datasets in real-time [5].

After the first implementations of HOT lanes in California [6], several studies had included evaluations and innovations of this concept [6,7,8,9,10]. Multiple research efforts have investigated the impact of sensor density (spacing) [11,12,13]. An older study analyzed the reliability and health of the installed sensors [14]. The same study attempted to determine the empirical rates of sensor failures and identified sensor malfunctions and data loss during transmission as the typical sources of errors. A recent effort made by the Nordic traffic authorities strategic research cooperation (NordFOU) [15] compared the accuracy of multiple types and models of sensors. We note here that all previous research focused on the accuracy of the utilized sensors with no explicit attention given to ELs facilities or their dynamic algorithms. Specifically, the relevant literature does not address the sensitivity of toll calculations concerning the accuracy and reliability of sensor data.

Therefore, this paper aims to bridge this gap in the existing body of knowledge by evaluating the impact of detection accuracy on the ELs toll calculation utilizing a dynamic pricing algorithm. The evaluation methodology was applied in a case study of the ELs implemented on Interstate-95 in Southeast Florida. The dynamic toll calculation algorithm implemented in the field is replicated to accurately mimic the fluctuations in the ELs dynamic tolls based upon the data reported by the sensors. The study designs and applies dozens of realistic scenarios simulating the erroneous sensor readings and failures and analyzes their impacts on the performance of the dynamic pricing algorithm. In each of the evaluated scenarios (with the erroneous data) the fundamental traffic flow relationships (i.e., speed vs. volume) are maintained, meaning that ‘healthy’ sensor data are fabricated. The presented paper makes the following research contributions:

1. A robust methodology is established to investigate the sensitivity of failed sensors and their erroneous data.
2. The dynamic pricing algorithm used on Interstate-95 ELs in Florida is described and replicated (with proper calibration and validation) in MS Excel software by using Visual Basic for Applications (VBA).
3. The individual impacts of the erroneous detection readings and completely failed sensors on the existing dynamic toll calculation algorithm and the resulting toll revenues of Interstate-95 ELs are quantified.
4. The combined impact of multiple failed sensors and erroneous sensor data on the existing dynamic pricing algorithm and toll revenues of Interstate-95 ELs are quantified.

To quantify the impact of sensor failure and erroneous sensor data, the presented methodology uses the traffic data and dynamic toll pricing algorithm implemented at the studied ELs sections. In the analysis, the sensor failures and erroneous data are simulated in two sets of scenarios. In the first scenario set, the sensors are subjected to consistent traffic volume and speed detection errors of 5, 10, 15, and 20% across the board, or a pre-set percentage of sensors is assumed to have failed. The second set of scenarios includes sensors that failed or generated erroneous data. Those sensors are randomly selected based on pre-set percentages of failed sensors and detection errors. The methodology and the associated analysis are implemented in a series of experiments designed for a case study of ELs on Interstate-95 in Florida, utilizing the traffic data and dynamic toll calculation algorithm provided by the Florida Department of Transportation (FDOT). To the best of the authors’ knowledge, the methodology used to perform the analysis in this study is unique and has not been previously presented. We also note that the presented methodology could be applied on any road congestion pricing scheme at various HOT and express lanes to investigate the sensitivity of toll computations relative to traffic sensor ‘health’ and detection accuracy.

## 2. Literature Review

The term ‘congestion pricing’ was introduced by English economist Arthur Pigouin in 1920 [16]. The first serious discussion about road congestion pricing, as a form of mitigating traffic congestion, was presented by Maurice Allais, who played a crucial role in establishing the first implementation of road congestion pricing in Singapore [17]. According to Lombardi et al. [18], congestion pricing is mainly applied on three scales: managed lanes, tolled facilities, and networks. HOT and express lanes are currently implemented in 11 states in the United States, including California, Florida, and Texas [19]. The toll on most of those ELs is primarily priced using dynamic pricing algorithms [20]. However, fully dynamic pricing is theoretical as fixed time-of-day tolls are still implemented when necessary [21,22]. In the past two decades, dynamic toll pricing algorithms outperformed fixed tolls in managing traffic congestion, which attracted researchers to develop many dynamic pricing schemes. This section presents the state of the art in dynamic toll pricing since the early stage of developing dynamic pricing algorithms in 2008 [23,24]. Furthermore, this literature review summarizes the most notable studies that addressed detection accuracy and reliability of traffic sensors similar to those used to price tolls on ELs.

### 2.1. Dynamic Congestion Pricing Algorithms

Yin and Lou [23] were among the first to propose dynamic pricing approaches (ramp metering control algorithm and route choice model). Zhang et al. [24] utilized the feedback control theory to develop a more adaptive dynamic pricing algorithm. Sheu and Yang [25] integrated a ramp control strategy with a dynamic pricing algorithm to better manage the dynamic freeway congestion. Applicable dynamic pricing algorithms on large-scale networks were proposed in [26,27,28].

Until 2009, most literature on dynamic pricing algorithms and all implemented algorithms in the field were based on historical traffic data. For instance, Minnesota’s toll on I-394 HOT lanes is usually updated every 3 min regardless of drivers’ route choices [10]. When a change in traffic density is detected (based on data reported from sensors installed along the ELs), the toll is increased or decreased (based on the traffic density of the past 3 min) according to a pre-defined traffic density delta table [10]. Lou et al. [29] built on their work in [23] to propose a self-learning approach that seeks to learn motorists’ willingness to pay and then determines the best pricing strategy to be used. Similarly, Zhang et al. [30] proposed a self-adaptive pricing approach that seeks to maintain reliable travel time on ELs, especially when GP lanes are congested. In a more complex effort than works in [23,29], Jin et al. [31] developed a pricing scheme to accommodate different types of lane-choice models.

Chung et al. [32] expanded the literature on congestion pricing by converting the dynamic pricing into a robust optimization to minimize the network-wide total travel cost considering the uncertainty of traffic demand. Moreover, Yang et al. [33] developed a convergent trial-and-error method to control congestion under ambiguous travel time and traffic demand. Zou et al. [34] proposed a pricing scheme that considers the throughput uncertainty resulting from the delay between the ingress and egress points. Other optimization techniques were widely covered in the literature. For example, Alkeder and AlRashidi [35] used a genetic algorithm to solve the pricing optimization problem, whereas Cheng et al. investigated a mean-variance optimization approach [36]. Moreover, Liu et al. [37] proposed speed-based mathematical programming with an equilibrium constraint model to maintain the traffic condition in the tolled cordon. Recently, Zhong et al. [38] used multi-objective Bayesian optimization to consider the land use aspect in their proposed pricing model.

To alleviate the complexity of dealing with models presented in [24,29,32], an integration effort of a macroscopic model (to represent dynamics of congestion) with an agent-based simulator (to reproduce travelers’ choices and heterogeneity) was made by Zheng at al. [39] to evaluate a variety of dynamic cordon pricing schemes. Moreover, Yang et al. [40] proposed a mathematical model which can be calibrated with ease to solve the dynamic pricing problem for distance-based toll collection systems. Some studies utilized the network (aka Macroscopic) fundamental diagram to develop area-based pricing schemes [41,42,43].

The framework developed by Hassan et al. [44] was among the earliest efforts to formulate the dynamic pricing problem that maximizes revenue explicitly. Another pricing strategy to maximize revenue while maintaining a desirable level of service on the ELs was made by Cheng and Ishak [45]. Unlike the work in [44,45], Lou [46] formulated his toll optimization to maximize the throughput, regardless of revenue, on both ELs and GP lanes while ensuring a free-flow speed on ELs as the constraint. The environmental aspect was also considered in the dynamic pricing optimization problem proposed by Friesz et al. [47]. They proposed tolling a set of links in their studied network to minimize vehicle emissions and travel time. Minimizing total delay was the goal of the dynamic pricing schemes proposed by [48,49]. Other flexible pricing approaches were introduced in [50,51,52] to minimize delay, maximize revenue, or achieve levels of specified combinations of delay and revenue.

Michalaka et al. [53] introduced an add-on for the traffic simulation software CORSIM to model multi-segmented ELs with various pricing strategies considering motorists’ lane choice between ELs and GP lanes. To perform various congestion pricing studies on the New York City (NYC) network, a virtual testbed for New York City was developed by He et al. [54] using an agent-based simulation ‘MATSim’, an open-source simulation toolkit coded in Java and developed by the Federal Highway Administration (FHWA) [54]. Pandey and Boyles [55,56] proposed simulation-based pricing schemes for multi-segmented ELs. A few other simulation-based optimization frameworks were introduced to dynamically determine toll pricing that maintains an acceptable level of service on the ELs [57,58,59].

Gardner et al. [60] included the population value of time (VOT) in the route choice process between the ELs and GP lanes. Xiao and Zhang [61] considered not only VOT but also the differences in commuters’ VOT to develop Pareto-optimal solutions considering pricing and subsidy in the dynamic pricing optimization problem. Several similar studies proposed pricing schemes that account for VOT heterogeneity between travelers [62,63,64,65].

Cheng et al. [66] proposed an agent-based model approach to dynamically determine tolls based on the travel time difference and travel time reliability between ELs and GP lanes. A similar idea was proposed by Cheng et al. [67] considering the actual time delay experienced by motorists combined with the traveled distance in the pricing scheme. A recent effort was made by Chen et al. [68] to consider people’s satisfaction (after using a tolled facility) to develop a pricing model.

The big data technologies and machine learning algorithms were also deployed to develop dynamic pricing schemes. For example, Figueiras et al. [20] developed a pricing scheme that depends on big traffic data technologies and aims to improve traffic flow and make toll prices competitive for various motorists. Liu and Yang [69] combined the travel and pricing data (information) to investigate motorists’ route choices (e.g., between ELs and GP lanes). Yang et al. [70] utilized connected vehicle data to develop a pricing scheme for large-scale areas. Several recent studies developed deep reinforcement learning frameworks for dynamic pricing on multi-segmented ELs [71,72,73].

### 2.2. Traffic Data Sensors

Detection accuracy and reliability have gained much attention from many researchers in the past two decades. This interest is attributed to the crucial role of traffic detection in managing and improving traffic performance. For example, one of the goals of traffic sensors on ELs is to capture the traffic conditions to accurately manage the toll pricing process. Since little research efforts were made on the accuracy of data detection on ELs, this subsection summarizes some of the most notable studies that addressed sensor detection issues relevant to those found in ELs.

Data aggregation intervals are vital, especially in dynamic pricing algorithms, as data-driven toll pricing is based on the accuracy of traffic conditions measured in real-time. Some authors addressed the general matter of the time intervals for archiving the data. For instance, Gajowski et al. [74] showed that aggregation levels of 60 min or more and less than 1 min are optimal for low and high traffic travel time estimation, respectively. Oh et al. [75] indicated that an archiving aggregation level of 4–6 min is desired when forecasting travel times. Park et al. [76] found that the optimal aggregation sizes for the travel time estimation and forecasting were 3 to 5 min and 10 to 20 min, respectively. Vlahogianni and Karlaftis [77] examined speed data aggregated in 20 s, 1, 5, 10, 15, 30, and 60 min. The results show that as the sample size increases, the aggregated data are less accurate (the relationship is not linear), and the optimal aggregation interval was 20 s during peak periods. Son et al. [78] tested traffic while transitioning from uncongested to congested conditions, with data aggregated in 15-min intervals. Those two conditions are significantly different and the speed difference was around 20 mph. However, the aggregated data did not capture this transition where traffic flow and speed were underestimated, especially in near-saturated conditions.

ELs operating agencies use different time intervals to update their tolls. For example, the most frequent toll update is in Minnesota. The MnPASS (the marketing name for a transportation service in Minnesota [10]) electronic toll collection system updates toll amounts every three minutes regardless of raw traffic data being obtained every 30 s [79]. In Texas, the toll amounts for TEXpress lanes are updated every five minutes [80]. FDOT implements a dynamic algorithm that updates toll amounts every 15 min [3]. No studies addressed how the frequency of toll updates affects the accuracy of defined tolls and traffic conditions.

A few studies have been conducted to document the impact of the spatial frequency of detection on the reported data accuracy. For instance, Chaudhuri et al. [11] studied the spacing of loop sensors and found that the spacing between sensors significantly impacts the accuracy of travel-time estimates. Fujito et al. [12] investigated the impact of sensor spacing on estimating traffic performance measures. They concluded that the actual sensor locations influence travel time estimation more than the spacing between them. Kwon et al. [13] suggested that placing sensors every half mile would reduce the errors in the average duration of congestion and the average spatial extent to 10%.

There are various vehicle detection systems and technologies that retrieve different traffic data types, all of which have pros and cons. Inductive loop sensors are used for various traffic applications, and they are the most commonly used systems for real-time data collection and toll collections [81]. For that reason, several studies validated traffic data from loop sensors by comparing it with traffic data collected using other methods. El-Geneidy and Bertini [82] compared speeds obtained from the Automatic Vehicle Location (AVL) technology to speeds reported by loop sensors. Tong et al. [83] and Chaudhuri et al. [11] validated speed detected data against speed data measured by GPS-equipped probe vehicles. Bar-Gera [84] compared speeds estimated using cellular phones as travel probes to loop sensor speeds.

According to Mimbela and Klein [81], two-loop speed traps are more convenient for express lanes and toll pricing than single loops because the latter do not predict speed accurately. Magnetic sensors can also be utilized in express lanes since they provide the primary traffic data [81]. However, in the last decade, Microwave Vehicle Detection Systems (MVDS) have been increasingly used for electronic toll pricing and collection [85]. The MVDS collect lane presence, volume, occupancy, and speed data [85]. FDOT and the Texas Department of Transportation (TxDOT) implement MVDS technology on their express lanes. They require a minimum of 90% accuracy for traffic lane presence, speed, and volume detection, and 85% accuracy for occupancy [86,87].

### 2.3. Summary

Researchers have made an enormous effort to develop dynamic pricing algorithms considering various aspects and objectives. However, little literature is available on traffic data sensors. Precisely, the relevant literature fails to address the sensitivity of toll calculations to traffic sensors’ reliability and accuracy. Thus, this study attempts to fill this gap by investigating the impacts of erroneous and failed vehicle detection on the quality of dynamic toll calculations.

## 3. Methodology

### 3.1. Study Site and Data Collection

The Southeast Florida ELs permit two-axle vehicles and public buses that are SunPass (the electronic toll collection system within the U.S. state of Florida) customers. Trucks with three or more axles are prohibited on the ELs unless they are designated as emergency vehicles. FDOT calculates the toll amount based on volumes and speeds from MVDSs on I-95 ELs, installed every half of a mile. The recorded MVDS speeds are estimated as point speeds, based on all vehicles’ speed measurements that cross the relevant MVDS zone during a particular 15-min interval. Although part of Florida’s toll collection system encompasses inductive loops that trigger the toll collection and enforcement cameras, those inductive loops are not used in the toll pricing algorithm [3]. Toll adjustments are used to maintain near free-flow speeds in the ELs. As traffic density in the ELs increases, the tolls increase, and vice versa. The current toll rates for the next three destinations/exits are shown on dynamic message signs (DMS) located in advance of the ELs’ entry points. If a toll increases after a driver enters the express lanes, the driver still pays only the toll that was shown on the sign when they entered the ELs. However, if the toll decreases after the driver’s entry, the driver is charged the lower amount.

#### 3.1.1. Data Collection and Analysis

The authors utilized 2017 dynamic toll data (provided by the FDOT) to select a segment and representative days for the study. The posted tolls were examined for six directional segments (three in each direction, Southbound and Northbound). The analysis of the toll data focused on quantifying toll fluctuations during various time intervals. Two segments of Interstate-95 (segments 2-South (2S) and 2-North (2N)) were selected for further analysis because they had the most toll fluctuations.

#### 3.1.2. Study Area

**Figure 1:**
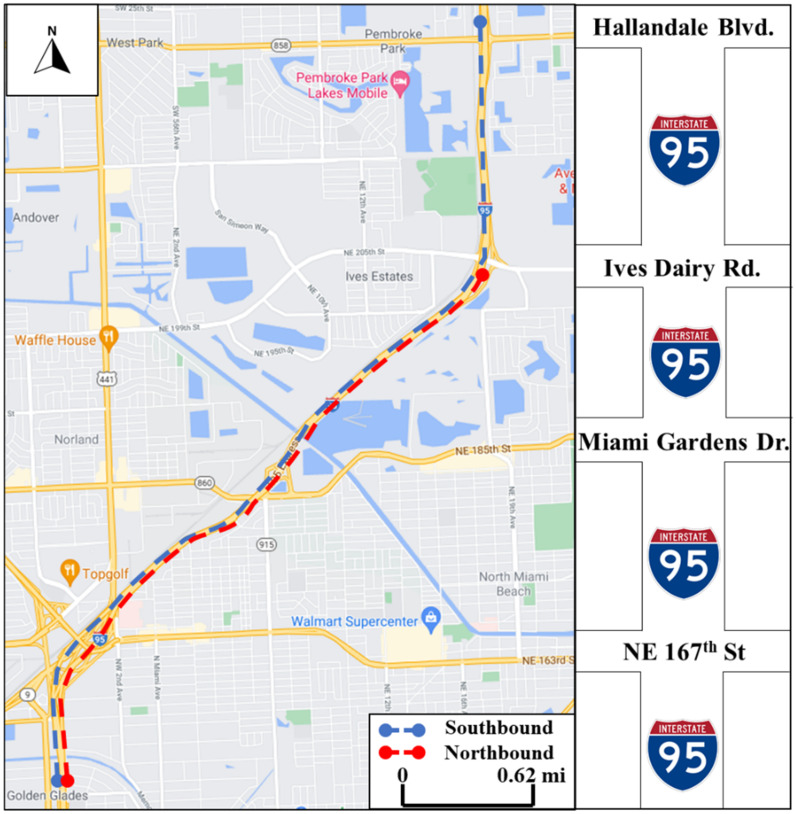
Study area of I-95 express lanes.

Figure 1 shows the study area. The Southbound segment is 6 miles long, from north of the Hallandale interchange to a point south of NE 167th Street. The Northbound segment is 4.5 miles long, from south of NE 167th Street interchange to the south of Ives Dairy Road in the north. Although several sensors are installed along with the tested segments, not all field sensors are used to retrieve traffic densities for toll calculations. The FDOT has an internal process by which it compares the data readings from the relevant MVDSs to those from gantry EL sensors to ensure that only reliable MVDSs are used for toll calculations.

#### 3.1.3. Selection of Representative Days

A data filtering process was performed for the studied segments for the entirety of 2017 to choose representative days, which were selected according to the following criteria:

- The tolling operations were not closed during any portion of the investigated period;
- The tolls do not reach the maximum value allowed by the algorithm (to avoid oversaturation conditions that can impact the results of the conducted experiments);
- The tolls exhibit smooth and noticeable toll changing patterns (that will ensure testing the sensitivity of sensors at various traffic ‘demand’ densities);
- The toll operations use the maximum number of sensors for toll calculations;
- The representative days experienced a close-to-an-average traffic volume in 2017.

**Figure 2:**
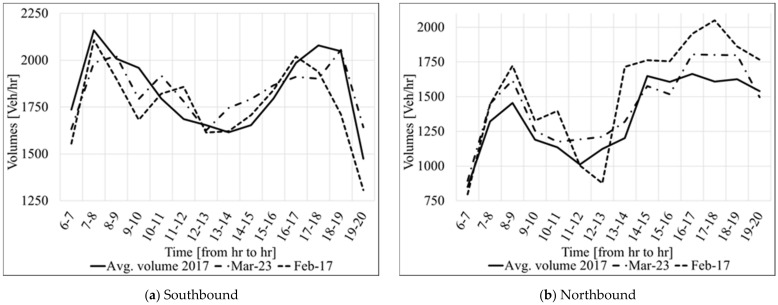
Average hourly traffic volume for 2017 and the representative days.

To meet all of the stated criteria, the raw volume data (20-s interval) were collected from the Regional Integrated Transportation Information System (RITIS) and analyzed to identify the representative days. The selection process started by excluding all days when ELs were closed (e.g., tolls were not charged) on the investigated ELs segments. Then, the remaining days were inspected according to the presented criteria. Although many days met the criteria above, 17 February 2017 and 23 March 2017, were selected as representative days for further analysis. On the one hand, 23 March 2017 was selected because it represents a close-to-an-average daily volume in 2017, as shown in Figure 2. However, on that day, an average volume on Northbound does not impact the toll amount significantly. Thus, 17 February 2017 was added to the analysis because it represents a day with relatively high traffic volume on NB (as shown in Figure 2b), which helps to evaluate the impact of the accuracy of sensors on the toll amount on the Northbound segment. It is worth noting that the directional distribution factors (D) for the two selected days are slightly different. 17 February had 53.41% of traffic flowing in the Southbound direction, whereas on 23 March this percentage was slightly higher (55.78%).

Multiple scenarios were designed to evaluate the impact of inaccurate detection on calculated tolls. Those scenarios were performed on a prototype of the existing algorithm used by the FDOT on I-95 EL to precisely imitate the algorithm used in the field. The traffic demand was structured in such a manner to replicate the field conditions closely. A customized spreadsheet tool was developed to replicate the FDOT algorithm and perform the experiments. In addition, carefully selected performance measures were utilized to assess the effect of inaccurate vehicle detection on calculated tolls.

### 3.2. Pseudocode for Dynamic Toll Module

The dynamic toll mode is applied on I-95 ELs to adjust toll rates based on detected real-time traffic conditions. Traffic density is used to determine the level-of-service (LOS) that reflects the condition of traffic flow. Irrespective of the toll amount calculated by the algorithm, each LOS is configured, within the algorithm, to have a minimum and a maximum toll rate amount. These minimum and maximum thresholds serve to limit the tolls charged to the motorists for a given LOS. For the reader’s convenience, Table 1 summarizes the notation used in this section.

**Figure 3:**
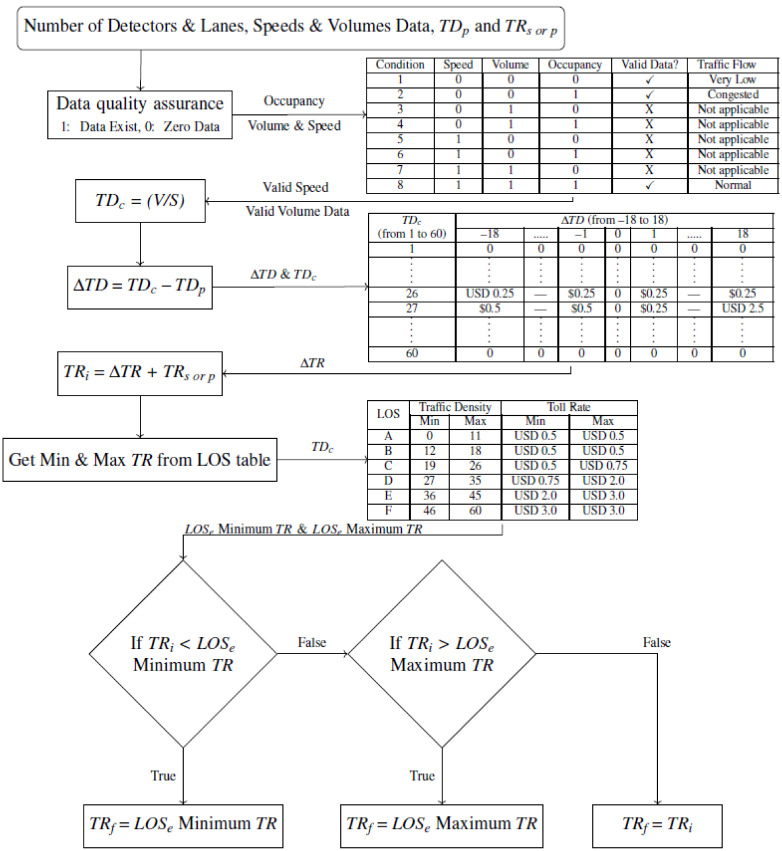
Toll calculation algorithm flowchart.

Although the FDOT algorithm can use either traffic densities or travel times as the primary measure to calculate tolls, it currently relies solely on the traffic-density method. The algorithm starts with a toll for the previous interval (or a seed value if the algorithm starts for the first time) and then changes that toll based on the traffic speed and volume data collected from the selected EL MVDS(s) for the preceding 15 min. The collected traffic speeds and volumes are ‘cleaned’ to exclude invalid data. The data inputs that meet the data quality assurance conditions 1, 2, or 8 (shown in the first table of Figure 3) are considered valid data.

Then, traffic density (veh/mile/ln) is calculated using the traffic link flow (based on traffic volumes) and traffic link speed, as shown in Equation (1). To obtain the traffic link flow, the total volume during a particular 15-min period is multiplied by 4 (Equation (2)) to find the equivalent hourly traffic volume for the same 15-min interval. For speed, an average speed for a specific 15-min period is computed by dividing the sum of all speeds (*n*) (each speed is an average of one vehicle’s speeds) collected during that 15-min period by the total number of vehicles from the same period, as shown in Equation (3).

(1) $$TD=\frac{F_{TL}}{S_{TL}\times NL}$$

(2) $$F_{TL}=\frac{V_{TLC}}{DCI}\times 3600=\;V_{TLC}\times 4$$

(3) $$S_{TL}=\frac{\sum_{i=1}^{n}\;s_{i}}{V_{TLC}}$$

The current and previous traffic densities and the traffic density delta table (the second table shown in Figure 3) are used to determine the EL toll adjustment to reflect the current traffic flow. The table covers various values of delta densities (−18 to 18 veh/mile/ln) and current densities (1 to 60 veh/mile/ln).

Afterward, the toll adjustment is added to the previously calculated toll to calculate an initial toll (based on traffic density). This initial toll is then compared to the minimum and maximum tolls that correspond to the existing LOS (LOSe Minimum TR and LOSe Maximum TR, respectively). Examples of the minimum and maximum tolls are given in the third table of Figure 3. If the initial toll rate is either smaller than the minimum toll or greater than the maximum toll, a new toll is set to minimum or maximum value, respectively. Otherwise, the initial toll becomes a new toll to be implemented and displayed to the drivers. Figure 3 uses the nomenclature presented in Table 1 to depict the above-explained process.

### 3.3. Scenarios for Modeling Detection Errors

As previously mentioned, not all field sensors are used to retrieve traffic densities for toll calculations. Thus, the data for the representative days were reviewed to determine which sensors were utilized for toll calculations on the representative days. The findings show that four sensors (labeled as SB014.3-DS-EL, SB014.6-DS-EL, 14.9-DS-EL, and SB015.1-DS-EL) were used to determine the toll values on the Southbound segment and three sensors (labeled as NB014.5-DS19-EL, NB014.6-DS-EL, and NB014.9-DS-EL) were used for the Northbound segment. To investigate the impact of the accuracy of vehicle detection on toll calculation, two sets of scenarios were developed:

- **Scenario 1**: Systematic detection errors—all sensors consistently generate erroneous readings with 5, 10, 15, or 20% error across the board. This scenario includes two subsets:
  - ***Subset 1*** consisted of a set of uniform detection errors for all of the sensors on the tested segment, of ± 5, ±10, ±15, and ±20% of the detection values observed in the field. For example, a +5% error in volume (with a corresponding error in speed and the resulting error in density) had been applied to all of the sensors on the studied segments by multiplying their observed field volumes with a factor of 1.05.
  - ***Subset 2*** consisted of a set of experiments where a certain percentage of sensors was considered to have failed completely (e.g., no reported traffic data), thus leaving the algorithm to rely only on the rest of the sensors (that have remained operational). Therefore, Subset 2 represents hypothetical field conditions when sensors either work or do not work—there is no error applied. However, various percentages of the available sensors were simulated to fail within the two analyzed EL segments.
    Table 2 lists all of the 25 experiments conducted under the Scenario 1 set, each with an associated percent of error or a percent of failed sensors. We note here that if 100% of sensors on a particular segment fail, it is assumed that the pre-designed tolls (from the day-of-week/time-of-day tables) are used.
- **Scenario 2**: Stochastic detection errors—the Monte Carlo simulation method was utilized to randomly introduce errors and failures of the sensors at the two ELs segments. All sensors within the studied segments had equal probabilities of falling into the group of failing sensors or their erroneous data. A custom stochastic model was developed to model the errors and failures throughout the analyzed ELs segments. The logic behind this approach was that some of the sensors could ultimately fail while others will either work with erroneous outputs or work properly. The objective was to investigate how such stochastically distributed detection errors and failures would impact the ELs toll calculations. Table 3 shows how stochastic experiments were selected and executed. The first column of Table 3 shows experiment ID, the second column shows the percentage of sensors set to fail in the corresponding experiment, the third column lists the sensors that were operational (with or without an error), the fourth column shows the level of error applied to (some of) the operational sensors, and the fifth column lists the operational sensors that were modeled with an error. The rest of the sensors in each experiment were assumed to be 100% functional and accurate.

### 3.4. Replication and Validation of the Toll Calculation Algorithm

**Figure 4:**
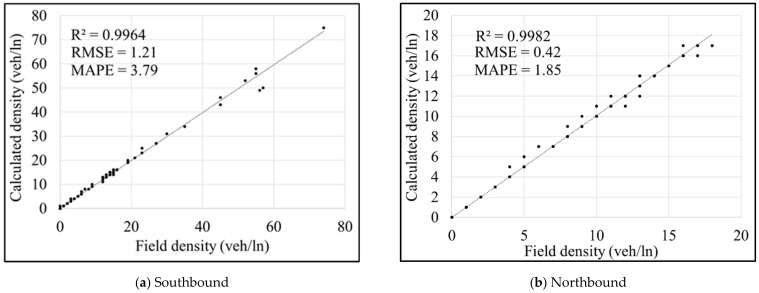
Results of density validation.

To simplify the process of running the experiments, the dynamic algorithm shown in Figure 3 was coded in MS Excel software using Visual Basic for Applications (VBA). The field data containing speeds, volumes, posted tolls, and traffic densities for the representative days were provided by the FDOT. The replicated FDOT toll calculation algorithm was validated by comparing traffic densities and posted tolls from the Excel spreadsheet (based on the raw field volumes and speeds) and those reported by the FDOT. Figure 4 shows the validation results for both Southbound and Northbound segments.

Based on the validation results, the field densities are well represented by the calculated densities, as demonstrated by low values of the root mean square (RMSE) and mean absolute percentage (MAPE) errors. Although insignificant differences (between the calculated and field densities) were found in the validation process, the calculated tolls were equal to the field-observed tolls (RMSE and MAPE equal to zero). That is due to the algorithm’s nature (shown in step “Get Min and Max TR from LOS table” in Figure 3), which ensures that small changes in densities do not impact the value of tolls.

### 3.5. Modeling of Traffic Density in Evaluated Detection Scenarios

When modeling errors, it was essential to take care of the effect that a change in specific traffic data (e.g., volumes) would impact the other data (e.g., speeds and density). In other words, it was necessary to account for interdependencies between the fundamental traffic flow characteristics (i.e., traffic volumes and speeds). To account for this phenomenon, the modified vehicular volumes (to model relevant errors) were used first to calculate the traffic density which was subsequently used to determine the toll rates. It was also necessary to estimate the average vehicular speed as a function of the given vehicular volume to determine traffic density.

**Figure 5:**
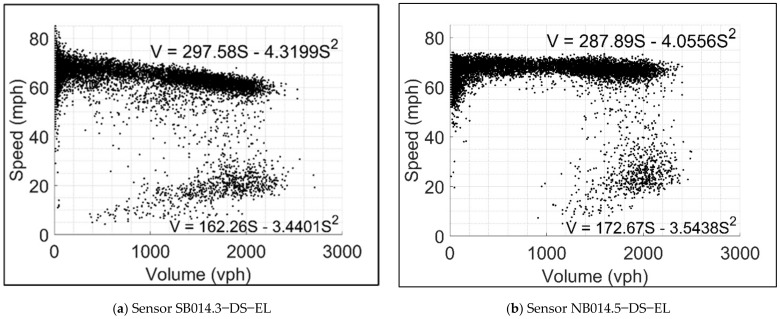
Speed-volume relationships from representative Northbound and Southbound sensors.

Considering that volume and speed are interdependent variables (one cannot be modified to represent the detection error without affecting the other), defining a relationship between speed and volume was necessary. The field volume and speed data were acquired from the sensors on both studied segments to accomplish that. A volume-speed (V-S) relationship was then calibrated for each sensor. Vehicle flow saturation conditions (i.e., undersaturated and oversaturated conditions) were also identified using custom-developed scripts in Python and MATLAB. Figure 5 shows a sample of the resulting V-S relationships for a pair of sensors (one in each studied direction). One can observe that the charts in Figure 5 suggest that some queueing occurred on the ELs, which contradicts the purpose of ELs for managing traffic congestion. However, it should also be noted that the calibrated relationships reflect a full year of various traffic conditions which likely include some heavy traffic incidents and the displayed oversaturated conditions.

The following steps outline (i) how errors were applied to the traffic volumes, (ii) how the corresponding speeds were calculated based on the V-S relationships, and (iii) how densities were calculated by using the volumes and speeds adjusted for an applied error:

1. Take the aggregated 15-min “true” volume and speed observations from the dataset, *V*1 and *S*1;
2. Multiply *V*1 by percent of error—representing an erroneous reading (+/− *Z*%) to obtain *V*2;
3. Insert *V*2 into the appropriate V-S relationship to obtain $\bar{S2}$ (the speed that corresponds to the adjusted volume, *V*2). A 40–50 mph speed range represents the queue discharge flow condition; thus, 45 mph is used as a threshold value to distinguish between undersaturated and oversaturated conditions.
4. Calculate speed error percentage by using the following equation:
  (4) $$F\%=\frac{\bar{S2}-S1}{S1}$$
5. Apply speed error *F*% on the true speed *S*1 to obtain *S*2;
  (5) $$S2=S1+S1\cdot \;\left(\frac{\pm F\%}{100}\right)$$
6. Use *V*2 and *S*2 to calculate 15-min traffic density (*D*):
  (6) $$D=\frac{V2}{S2}$$

### 3.6. Performance Measures to Evaluate the Impact of Detection Errors

Defining and quantifying the appropriate performance measures is critical in evaluating the performance of a dynamic ELs toll pricing algorithm. For this purpose, four performance measures (p.m.s) were selected in this study to assess the effect of different detection error scenarios on calculated traffic densities and tolls.

1. Mean absolute percentage error (*MAPE*), shown in Equation (7), was used to assess density variation in different scenarios. Equation (7) has three terms: *TTD* is true traffic density, which is the field-measured density, *CTD* is calculated traffic density after applying a percent of error to volume and speed readings, *n* is the number of 15-min intervals used in the calculation, and *i* is any 15-min interval.
  (7) $$MAPE=\left(\frac{100\%}{n}\right)\overset{n}{\underset{i=1}{\sum }}\|\frac{TTD_{i}\;–\;CTD_{i}}{TTD_{i}}\|$$
2. Absolute toll error (*ABS*) is important for observing the cumulative total absolute change in tolls for all 56 15-min intervals during the study period (6 a.m.–8 p.m.). Equation (8) shows how *ABS* is computed where true toll rate (*TTR*) represents the posted toll in the field, whereas calculated toll rate (*CTR*) is taken from the experiments, and *i* is any 15-min interval.
  (8) $$\mathrm{ABS}\;15-\;\min\;\mathrm{toll}\;\mathrm{error}=\overset{n}{\underset{i=1}{\sum }}\|TTR_{i}-CTR_{i}\|$$
3. Total gross toll: a sum of the products of volumes of the charged vehicles and their tolls for each of the 15-min intervals. The total gross toll represents the total toll fees calculated for each scenario during the analysis period (6 a.m.–8 p.m.). It is crucial to mention here that all the total gross toll values are theoretical and not actual. By using “i” for any 15-min interval during the analysis period, the following equation defines the total gross toll:
  (9) $$\mathrm{Total}\;\mathrm{gross}\;\mathrm{toll}\;=\overset{n}{\underset{i=1}{\sum }}\left[TV_{i}\cdot \;CTR_{i}\right]$$
  where: *TV* is true volume, and *CTR* is calculated toll rate.
4. Equation (10) computes the difference in total revenue between the true total gross toll charged in the field and the total gross toll calculated in the experiments with erroneous detection. This measure helps to see the impact of detection errors or failures on the total gross toll.
  (10) $$\mathrm{Difference}\;\mathrm{in}\;\mathrm{total}\;\mathrm{revenue}\;=\overset{n}{\underset{i=1}{\sum }}\left[TCR_{i}-TAR_{i}\right]$$
  where: *TCR* is total calculated revenue, *TAR* is total actual revenue, and *i* is any 15-min interval.

In Equation (10), a negative value means that toll revenue generated due to erroneous detection is lower than the revenue during normal conditions (with no detection errors or failed sensors). On the other hand, a positive difference means that erroneous detection would result in extra tolls charged to the travelers at a given time.

## 4. Results and Discussion

**Figure 6:**
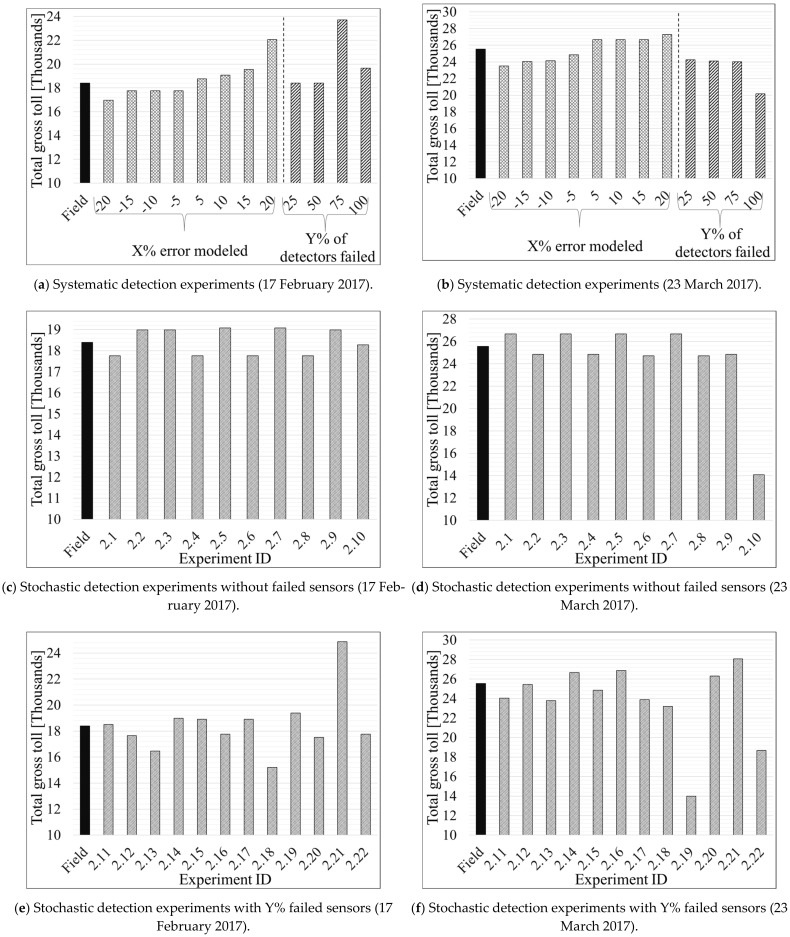
Graphical interpretation of total gross toll for systematic and stochastic failure and error scenarios on Southbound direction.

**Figure 7:**
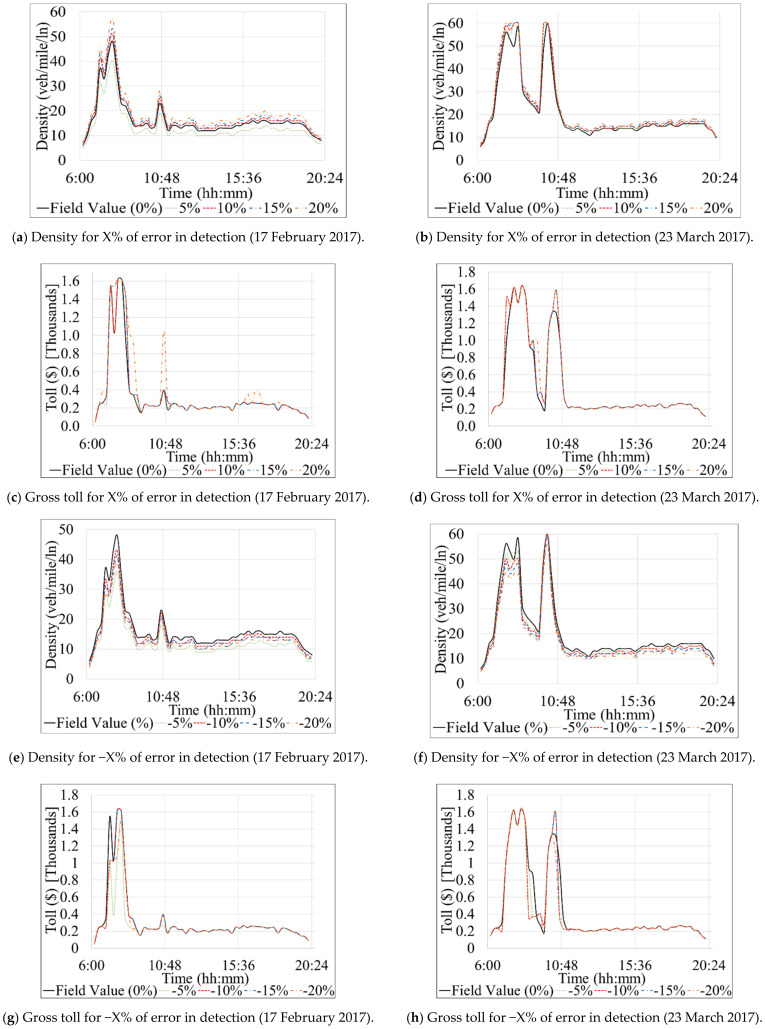
Diurnal fluctuations of density and 15-min gross tolls on Southbound direction.

**Figure 8:**
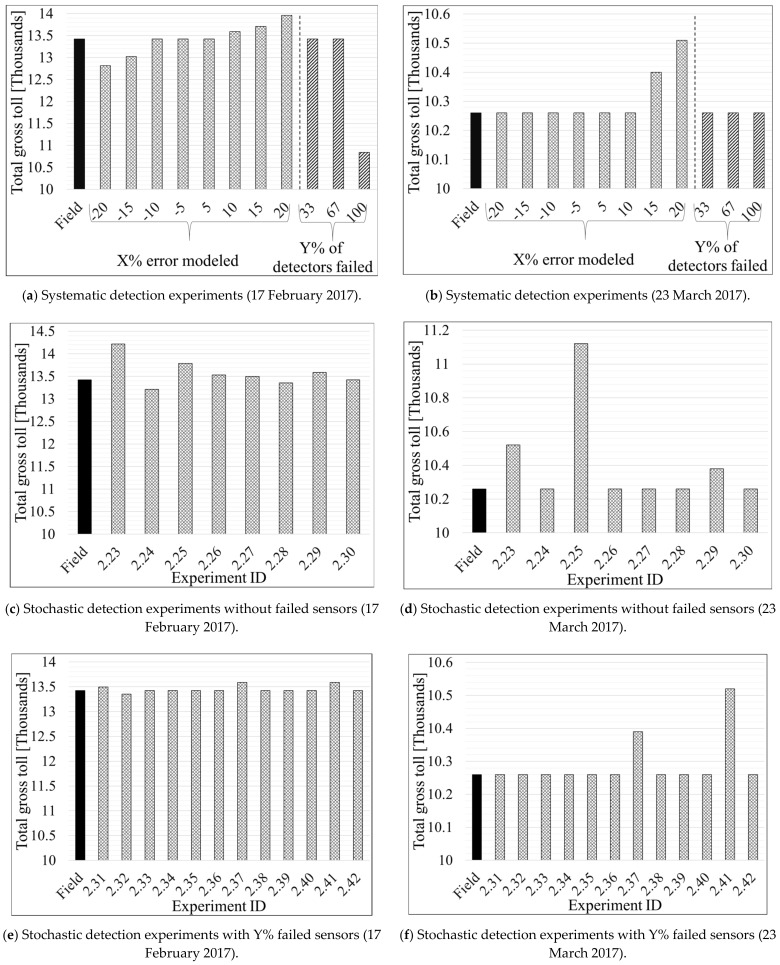
Graphical interpretation of total gross toll for systematic and stochastic failure and error scenarios on Northbound direction.

**Figure 9:**
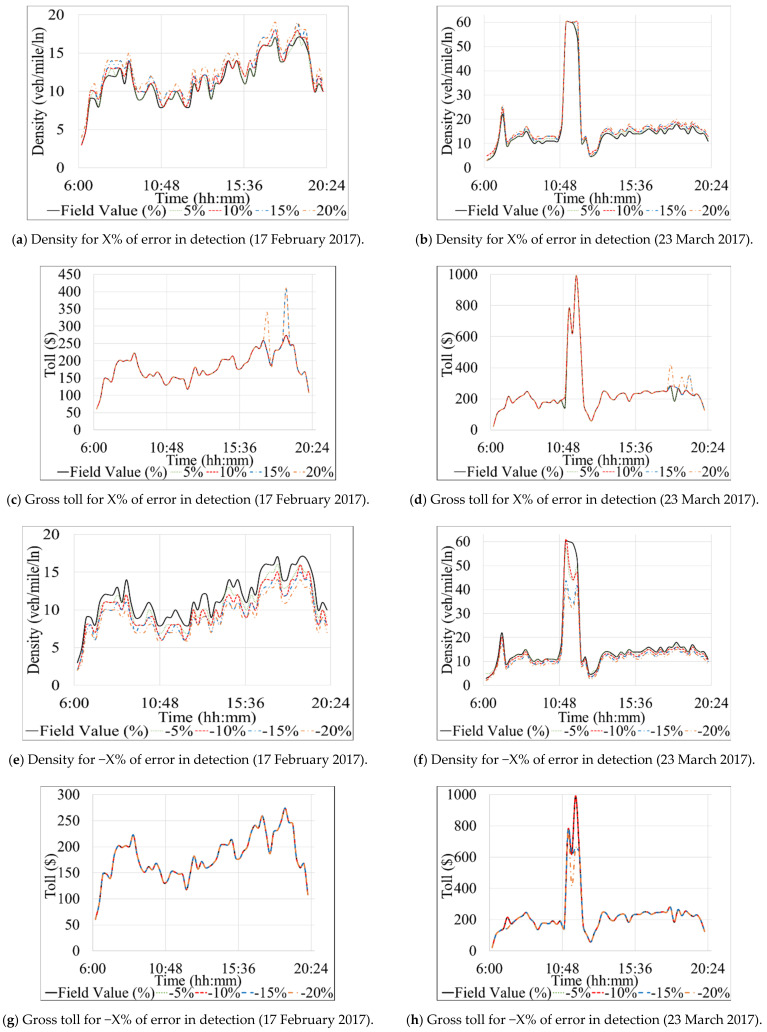
Diurnal fluctuations of density and 15-min gross tolls on Northbound direction.

Table 4 and Table 5 present the results of Scenario 1 and 2 for the Southbound direction. Similarly, Table 6, Table 7, and Table 8 show the results for the Northbound direction. The profit and loss values, shown in the last column of each table, were computed by subtracting a specific experiment’s total toll value from the total field toll where no error or failures were modeled (Equation 10). Figure 6 depicts the total gross toll for the Southbound direction computed based on the 15-min toll values derived for each experiment and the corresponding traffic volume during each 15 min of the studied period (6 a.m.–8 p.m.). Figure 7 shows changes in density and 15-min gross toll for all experiments performed under subset 1 of Scenario 1 for the Southbound direction. Figure 8 and Figure 9 are similar to Figure 6 and Figure 7, respectively, but for the Northbound direction.

### 4.1. Analysis of the Southbound Segment

#### 4.1.1. Scenario 1: Systematic Detection Errors

Table 4A shows the results of applied measures for the experimental subset 1 of the systematic detection errors for the Southbound direction (introduction of detection error of ±5, 10, 15, or 20%, respectively, for all sensors on the segment). This subset consisted of nine experiments, as shown in Table 4A. The left sections of Figure 6a,b show the total change in gross toll for the representative days of 23 March and 17 February, respectively, during the time of interest (6 a.m.–8 p.m.).

As expected, Table 4A and the negative percentages in the left sections of Figure 6a,b show that all of the experiments where traffic volumes were systematically underestimated, due to modeled detection errors (experiments #1.3, #1.5, #1.7, and #1.9), resulted in a loss of the total gross toll on both of the tested representative days. The toll revenue loss on 17 February 2017, was as low as USD 631 for systematic errors of −5, −10, −15% modeled for volumes from all sensors, whereas the loss reaches USD 1422 when the modeled error is −20%. On the other hand, loss on 23 March 2017 ranged from USD 710 to USD 2035 for modeled errors of −5 and −20%, respectively. Such a high difference between the loss on the two studied days is attributed to the relatively higher traffic volume on the Southbound section on 23 March 2017 compared to 17 February 2017. One can observe from Table 4A that the maximum change in density occurred when sensors reported (uniformly) with an error of −20% (experiment #9) in traffic volume. This magnitude of the error would change overall density estimation by around ~19%, which would result in a theoretical toll error of USD 4.75, or a loss of total gross toll of USD 1422 and 2035.75 for 23 March and 17 February, respectively.

**Table 4:** Results of systematic detection error scenario for the Southbound segment.

(A) Systematic Detection Experiments—Subset 1								
Exp. ID	MAPE (Density)		Absolute 15-min Toll Error (USD)		Total Gross Toll (USD)		Profit/Loss (USD)	
	17 February	23 March	17 February	23 March	17 February	23 March	17 February	23 March
1.1	0	0	0	0	18,394	25,548	0	0
1.2	5.85	4.79	1.25	2.25	18,752	26,667	358	1118
1.3	4.17	6.5	5.75	3.5	17,763	24,838	**631**	**710**
1.4	6.97	6.54	1.5	2.25	19,075	26,667	681	1118
1.5	9.05	10.2	1.25	5	17,763	24,135	**631**	**1413**
1.6	10.34	9.39	2.5	2.25	19,588	26,667	1194	1118
1.7	13.24	14.56	1.25	4.75	17,763	24,040	**631**	**1507**
1.8	19.44	12.04	7.5	3.5	22,061	27,297	3667	1748
1.9	18.89	18.89	4.75	4.75	16,972	23,297	**1422**	**2035**
**(B) Systematic Detection Experiments—Subset 2**								
1.19	2.22	3.92	4.75	4.75	18,394	24,235	0	**1312**
1.20	2.22	3.92	0	5.00	18,394	24,120	0	**1428**
1.21	3.07	6.05	12.25	6.25	23,722	24,019	5328	**1529**
1.22	23.62	23.62	13.00	13.00	19,659	20,170	1266	**5378**

Loss, all results are based on simulated or hypothetical scenarios.

In contrast, the overestimated volumes increased the total gross tolls (as shown in experiments #1.2, #1.4, #1.6, and #1.8 in Table 4A and the positive percentages in the left sections of Figure 6a,b. Unlike experiments #1.3, #1.5, and #1.7 for 17 February 2017, which resulted in equal loss despite underestimating volumes by various percentages, the toll increased for each increment of the volume overestimation starting from USD 358 for a 5% modeled error on 17 February 2017, to a maximum of USD 3667 for a modeled error of 20%. The opposite case can be seen for 23 March 2017, where the positive modeling error increased the total gross toll by USD 1118. That was identical for all modeled errors except 20%, which increased the toll by USD 1748. It can be concluded that higher traffic volumes are more sensitive to negative detection errors and less sensitive to positive ones.

For 17 February 2017, Figure 7a,e shows the diurnal changes in density due to detection errors modeled under experiments of subset 1 of the systematic detection experiments. Similarly, Figure 7b,f show the change in density for the same subset for 23 March 2017. The charts (Figure 7a,b,e,f) show that the density increased and decreased, as expected, when the volume was overestimated and underestimated, respectively. It can be seen that the periods with high densities (e.g., the peak period between time 6:00–10:48 on Figure 7a,b,e,f) have experienced the most drastic increase or decrease (depending on the experiment performed). That is because the overestimation or underestimation of reported traffic volumes was performed as a percentage of the volume being manipulated.

Figure 7c,d present the difference in total gross toll resulting from the change in density between the actual field values and experiments with overestimated traffic volumes for 23 March and 17 February, respectively. One can observe from Figure 7c,d that the difference occurs mainly during the morning peak (6:30–10:45 a.m.). The similar phenomena, but with the reduction in the total toll, can be observed in Figure 7e,f. Interestingly, some exception to the expected results (i.e., higher volumes lead to higher density higher toll) can be seen in various charts. For instance, Figure 7h shows that between 9:26–10:26 a.m. the toll was increased while the volume was underestimated. That is due to the increase in speed after applying the relevant Volume-Speed relationship, explained in Section 3.5. It can be observed that a change in density at low traffic densities (e.g., below 20) does not impact the toll values as significantly as changes at higher traffic densities (e.g., greater than 25), which is intuitive. However, it is noticeable that the toll does not fluctuate as much as density in Figure 7 due to toll limitations imposed for various LOSs values, as shown in the third table in Figure 3, where a range of density corresponds to a range between the minimum and maximum tolls.

Table 4B and the right sections of Figure 6a,b depict the losses when specific percentages (25, 50, 75, and 100%) of utilized sensors systematically fail (as shown in experiments #1.19, #1.20, #1.21, and #1.22, respectively). It can be seen from experiments #1.19 and #1.20 in Table 4B and the 25 and 50% columns in Figure 6a that omitting the data reported from up to two sensors did not impact the total toll amount on 17 February 2017. However, utilizing only one sensor (experiment #1.21 in Table 4B and the 75% column in Figure 6a) led to an increase of USD 5328 in the toll on 17 February 2017. Whereas using the “day-of-week/time-of-day” tables (no sensors) increased the tolls by USD 1266, as shown in experiment #1.22 in Table 4B and the 100% column in Figure 6a.

In marked contrast, the analysis for 23 March 2017 shows that omitting the reported data from one sensor resulted in a USD 1312 loss compared to the actual field total toll that should be collected (experiment #1.19 in Table 4B and the 25% column in Figure 6b). The loss increased when fewer sensors were utilized to compute the toll, as shown in experiments #1.20 and #1.21 in Table 4B and the 50 and 75% columns in Figure 6b. The maximum loss occurred when all sensors failed, in which case the “day-of-week/time-of-day” tables are used to determine tolls (experiment #1.22 in Table 4B) and the 100% column in Figure 6b. This result, accompanied by the result of using “day-of-week/time-of-day” tables on 17 February 2017, may suggest that the “day-of-week/time-of-day toll” tables are outdated. In other words, such tables were developed for traffic densities that no longer represent the studied ELs segment. It should be noted here that all the total gross tolls are compared to the gross total toll calculated from the field data (experiment #1.1 in Table 4A).

We note that scenarios where 25, 50, or 75% of sensors fail are not unrealistic in the field, as each segment contains a few sensors. Thus, if a single sensor fails, this may constitute 25–33% of all sensors available on that segment. It is worth noting that experiments #1.19 and #1.20 represent actual scenarios from the field where only three and two sensors, respectively, were used for toll calculations for four months in 2017. However, the authors do not report any actual losses or increase in the total toll during that period as no volumes or speeds data were provided from any sensor for those four months.

#### 4.1.2. Scenario 2: Stochastic Detection Errors

Table 5 presents various performance measures of each experiment conducted for the scenario with stochastic detection errors. To remind the reader, the stochastic detection errors scenario included combinations of erroneous sensor detection and either a fully functional or failed sensors. It can be observed in Table 5 that the tolls on the Southbound segment are highly responsive to the significant changes in sensor inputs caused by the emulation of erroneous readings (experiments #2.1 through #2.22 in Table 5 and Figure 6c–f). The results of stochastic experiments #2.1 through #2.10 reiterate some of the findings from the experiments with systematic detection errors. The total gross toll increases when the applied error increases traffic volume/density and vice versa. However, this general finding was not seen in all experiments; hence more discussion is provided below for experiments performed under similar conditions.

Scenarios with no failed sensors (experiments #2.1 through #2.10 in Table 5 and Figure 6c,d) can be classified according to the number of sensors with modeled error into three categories. Experiments #2.1–#2.4 with two sensors, experiments #2.7 and #2.8 with three sensors, and experiments #2.9 and #2.10 with only one sensor reporting erroneous data. We note here that random error percentages were modeled stochastically on some of the utilized sensors in each of the experiments mentioned above (#2.1–#2.22 in Table 5), as previously presented in Table 3. Within this classification, we first discuss the results of the experiments that had the same number of sensors with either positive or negative modeled error. Then we move to draw a general conclusion on all experiments #2.1–#2.10.

**Table 5:** Results of stochastic detection scenario for the Southbound segment.

Exp. ID	MAPE (Density)		Abs 15-min Toll Error (USD)		Total Gross Toll (USD)		Profit/Loss (USD)	
	17 February	23 March	17 February	23 March	17 February	23 March	17 February	23 March
2.1	18.99	3.48	15.75	2.25	17,763	26,667	**631**	1118
2.2	16.47	5.06	15.25	3.5	18,983	24,838	589	**710**
2.3	16.68	2.98	15.25	2.25	18,983	26,667	589	1118
2.4	18.42	5.52	15.75	3.5	17,763	24,838	**631**	**710**
2.5	16	4.52	15	2.25	19,075	26,667	681	1118
2.6	19.46	9.27	15.75	3.75	17,763	24,722	**631**	**825**
2.7	15.9	5.9	15	2.25	19.075	26,667	681	1118
2.8	20.89	8.78	15.75	3.75	17,763	24,722	**631**	**825**
2.9	16.56	5.59	15.25	3.5	18,983	24,838	589	**710**
2.10	18.05	71.28	16.75	4.5	18,276	24,092	**118**	1426
2.11	17.08	32.15	16.25	4.75	18,507	24,040	113	1507
2.12	20.57	7.01	16	4.25	17,648	25,439	**746**	**109**
2.13	24.76	14.65	18.25	4.75	16,472	23,780	**1922**	**1768**
2.14	17.79	8.98	15.25	2.25	18,983	26,667	589	1118
2.15	17.6	6.32	15.5	5.5	18,907	24,401	513	**696**
2.16	21.63	13.3	15.75	5.5	17,763	10,852	**631**	**1655**
2.17	17.82	21.07	15.5	4.75	18,907	23,893	513	1324
2.18	26.85	16.48	20.75	6	15,205	23,192	**3189**	**2355**
2.19	17.68	71.88	14.5	23.75	19,383	13,999	989	**11,549**
2.20	21.74	7.19	16.25	4.5	17,520	26,323	**874**	775
2.21	20.32	11.52	13.25	5.5	24,866	28,056	6473	2507
2.22	21.63	56.08	15.75	14	17,763	18,682	**631**	**6866**

Loss, all results are based on simulated or hypothetical scenarios.

Experiments #2.1 and #2.3 had one mutual sensor (#14.6) with modeled error out of the two sensors used in both experiments. As expected for experiment #2.3, the overestimation of volumes resulted in a total toll increase of USD 589 and 1118 for both days, 23 March and 17 February, respectively, as shown in Table 5 and Figure 6c,d. Although the same result can be seen for experiment #2.1 on 23 March (increase by USD 1118), the result of 17 February indicates a total gross toll loss of USD 631 (experiments #2.1 Table 5 and Figure 6d). This interesting result contradicts the results of the systematic scenarios (Table 4), where positive modeled errors, individually, always led to higher total gross toll. Such finding can be attributed to the fact that, unlike Scenario 1 where errors were modeled for all sensors, in Scenario 2, sensors were not exposed to any modeled error which could have impacted the results. Similar patterns can be seen in the results of experiments #2.2 and #2.4, but with opposite signs (decrease in toll instead of an increase and vice versa), as shown in Table 5 and Figure 6c,d.

The findings from experiments #2.5–#2.8 are more stable and expected. Whenever a positive error was modeled, an increase in the total toll was observed and vice versa. We note here that the respective increase and decrease in total gross toll had the same value for each pair of experiments (#2.5, #2.7) and (#2.6, #2.8), which can be seen in Table 5 and Figure 6c,d. That might be because experiments #2.5–#2.8 had the same sensors (#14.3, #14.6, and #15.1) modeled with errors. Finally, the last group of the experiments with no failed sensors, experiments #2.9 and #2.10, had the same percentage of modeled error (−15% for experiment #2.9 and +15% for experiment #2.10) on the same sensor (# 14.9). The pattern of the results of those two experiments is consistent with the patterns found in experiments #2.1–#2.4.

One general observation from experiments #2.1–#2.10 (Table 5 and Figure 6c,d) is that the increase or decrease in the total gross toll is within a relatively small range: between USD −631 to681 and −710 to1426 for 23 March and 17 February, respectively. That does not mean that such differences are insignificant, but they could be much larger, as observed in some of the experiments for Scenario 1 with systematic sensor failures and/or detection errors (Table 4B). That may also suggest that the number of sensors used for toll computations is more important than the error in the reported data from those sensors. However, this finding cannot be generalized based on the experiments performed in this study only, and further research is needed to confirm this finding.

Experiments with both failed sensors and modeled errors on one or more of the utilized sensors (Table 5 and Figure 6e,f) can be classified based on the total number of sensors and number of sensors with modeled errors into five groups: experiments #2.11–#2.12 with a total of three sensors of which two sensors were exposed to modeled errors; experiments #2.13–#2.14 with all of the three utilized sensors having modeled errors; experiments #2.15–#2.16 with two utilized sensors including one with errors; experiments #2.17–#2.18 with a total of two sensors, both with modeled errors; and finally experiments #2.19–#2.22 with one sensor used and exposed to modeled error. We note here that although some experiments used the same number of sensors and some others even had the same number of sensors with modeled errors, a variety of different percentages of modeled errors were randomly applied in all experiments #2.11–#2.22.

Starting with experiments #2.11 and #2.12, those experiments showed expected results where overestimation of volumes led to an increase in total gross toll, and vice versa. Thus, it seems that omitting the data from one sensor in those two experiments did not impact the results significantly. The results become a bit erratic when observing the results of experiments #2.13 and #2.14. That is because negative modeled errors increased the total gross toll and vice versa, which is unexpected. That may suggest, as noted earlier, that the number of sensors utilized for toll computation plays a considerable role in the final toll value. The rest of the experiments’ results (experiments #2.15–#2.22) presented in Table 5 and Figure 6e,f seem to follow the general expected pattern with loss resulted from negative modeled errors and fewer utilized sensors, and vice versa. What is clearly noticeable is the positive correlation between the number of deployed sensors and the significant differences between the actual total gross toll that should be charged in various scenarios. Most experiments showed larger profit/loss from experiments with fewer sensors than those with the same number of sensors as deployed in the field, but with modeled errors, as shown in Table 4 and Table 5 and Figure 6. That again might suggest that the number of sensors is more critical for toll computation than the accuracy of the data reported by individual sensors.

### 4.2. Analysis of the Northbound Segment

#### 4.2.1. Scenario 1: Systematic Detection Errors

Table 6A and Figure 8a,b show the results of the first subset of the systematically modeled errors in the Northbound section. Before commencing the discussion, we recall that traffic volume on the Northbound on 17 February 2017 is slightly higher than the average volume in 2017. In contrast, traffic volume on 23 March 2017 is closer to the average volume. A few general observations can be drawn from Table 6A regarding experiments #1.10–#1.18. First, the most remarkable change in traffic density is seen with absolute modeled errors higher than 15%. Second, absolute modeled errors of less than 10% did not impact the total gross toll. Third, negative modeled errors (underestimated volumes) did not affect the total gross toll on 23 March 2017. Fourth, the resulting profit/loss range of USD −609 to 536, is lower than what is observed for the Southbound section (Table 4A). Fifth, tolls on 17 February 2017 were impacted by the modeled errors more than the tolls on 23 March 2017 because the former had higher traffic volumes than the latter. The following paragraph describes the results of each of the studied days specifically.

In Table 6A and the left section of Figure 8a it can be observed that the changes in total gross toll on 17 February 2017 resulted from modeling errors of −15 and 15% are not equal in magnitude (experiment #1.15 and #1.16). Those two experiments decreased and increased the total gross toll by USD 403 and 283, respectively. A similar note can be made for modeled errors of −20 and 20%, with a decrease and increase in the total gross toll by USD 609 and 536, respectively. One can conclude that a modeled error of +K% does not compensate for an equal modeled error with an opposite sign (e.g., −K% in this case, assuming that K is constant).

**Table 6:** Results of systematic detection error scenario for the Northbound segment.

(A) Systematic Detection Experiments—Subset 1								
Exp. ID	MAPE (Density)		Absolute 15-min Toll Error (USD)		Total Gross Toll (USD)		Profit/Loss (USD)	
	17 February	23 March	17 February	23 March	17 February	23 March	17 February	23 March
1.10	0	0	0	0	13,425	10,264	0	0
1.11	4.24	10.8	0	0	13,425	10,264	0	0
1.12	4.79	10.22	0	0	13,425	10,264	0	0
1.13	13.94	4.67	0.5	0	13,590	10,264	165	0
1.14	9.18	14.79	0	0	13,425	10,264	0	0
1.15	11.33	9.05	0.75	0.25	13,708	10,401	283	137
1.16	16.13	18.65	1.25	0	13,023	10,264	**403**	0
1.17	14.45	12.31	1.25	0.5	13,961	10,514	536	250
1.18	20.31	23.13	2.25	0	12,816	10,264	**609**	0
**(B) Systematic Detection Experiments—Subset 2**								
1.23	0.48	1.48	0	0	13,425	10,264	0	0
1.24	1.48	1.67	0	0	13,425	10,264	0	0
1.25	14.14	14.14	0	0	10,844	10,264	**2581**	0

Loss, all results are based on simulated or hypothetical scenarios.

On the other hand, and similar to the corresponding results for the Southbound direction, the tolls on 23 March 2017 are impacted only if the volume is systematically overestimated by 15% or more (experiment #1.15 and #1.17 in Figure 8b). However, even then the difference in the total gross toll is relatively small. The reason for such results can be found in the values of traffic density, which must be at least equal to 26 veh/mile/ln to trigger a change in toll value (as shown in Table 7); on 23 March 2017, this is only achieved when the traffic is overestimated by 15% or more.

Similar to Figure 7 for the Southbound case, Figure 9a,b,e,f illustrate how various error experiments resulted in different traffic densities for the Northbound segment but eventually had matching tolls most of the time as compared to the tolls that should actually be charged considering the true traffic volume (Figure 9c,d,g,h). A couple of instances during the evening peak period (Figure 9c,d) were the exception. The reason for such slight differences in tolls can be explained by the minimum and maximum thresholds used by the algorithm to constrain the range of possible tolls for a given LOS (Table 7). Specifically, the “Get Min and Max TR from LOS table” step illustrated in Figure 3 shows that a minimum traffic density of 26 (veh/mile/ln) is required to trigger a change in the toll amount, as mentioned previously. The constraints imposed by the minimum and maximum toll thresholds (for each LOS) explain why there are no toll changes for any detection errors that result in underestimated traffic volumes on 23 March 2017, as well as those that overestimate traffic volume by only 5 or 10%.

**Table 7:** Level of service and toll amount for the Northbound segment.

Level of Service	Traffic Density [Veh/Mile/ln]		Toll Amount (USD)	
	Minimum	Maximum	Minimum	Maximum
A	0	11	USD 0.50	USD 0.50
B	12	18	USD 0.50	USD 0.50
C	19	26	USD 0.50	USD 0.75
D	27	35	USD 0.75	USD 2.00
E	36	45	USD 2.00	USD 3.00
F	46	60	USD 3.00	USD 3.00

The results of the second subset of the systematic detection error experiments for the Northbound segment are presented in Table 6B and the right sections of Figure 8a,b. Experiments #1.19 and #1.20 showed that all experiments with fewer sensors (1–2 failed sensors) do not result in high enough densities to trigger toll changes. However, experiment #1.25, where all three sensors on the Northbound were set to fail, shows that the total gross toll from the time-of-day tables was equivalent to those calculated based on the field data on 23 March 2017. In contrast, a loss of USD 2581 is observed in the equivalent analysis for the 17 February 2017 due to higher volume on that day (Table 6B). This finding suggests that the “day-of-week/time-of-day” tables seem to be relatively correct for a day with average traffic volumes on the Northbound segment.

#### 4.2.2. Scenario 2: Stochastic Detection Errors

Table 8 presents the results of the random detection errors scenarios for the Northbound segment. Contrary to the results for the Southbound segment, experiments #2.23, #2.25, #2.29, #2.37, and #2.41 show that Scenario 2 with stochastic detection errors, presented in Table 8 and Figure 8c–f), cause insignificant changes (USD < 1000) of the total gross tolls. That is also true for high traffic volumes on the Northbound segment, as shown in the results for 17 February 2017 in Table 8 and Figure 8c,e. A general conclusion can be drawn based on the experiments with stochastic detection errors: the dynamic toll calculations are not very responsive to small fluctuations of density on the studied Northbound segment of the Interstate-95 ELs.

**Table 8:** Results of stochastic detection scenario for Northbound segment.

Exp. ID	MAPE (Density)		Abs 15-min Toll Error (USD)		Total Gross Toll (USD)		Profit/Loss (USD)	
	17 February	23 March	17 February	23 March	17 February	23 March	17 February	23 March
2.23	1.1	20.23	2.75	0.5	14,216	10,524	791	260
2.24	1.25	23.35	1	0	13,216	10,264	209	0
2.25	0.32	24.04	1.25	1.75	13,784	11,118	359	854
2.26	95.37	95.25	0.25	0	13,534	10,264	72	0
2.27	0.63	9.99	0.25	0	13,497	10,264	72	0
2.28	0.16	9.52	0.25	0	13,354	10,264	72	0
2.29	0.39	13.25	0.5	0.25	13,590	10,377	164	113
2.30	0.16	11.15	0	0	13,425	10,264	0	0
2.31	0.19	18.03	0.25	0	13.497	10,264	72	0
2.32	0.32	18.03	0.25	0	13,353	10,264	72	0
2.33	0.24	11.15	0	0	13,425	10,264	0	0
2.34	0.49	10.74	0	0	13,425	10,264	0	0
2.35	0.65	12.32	0	0	13,425	10,264	0	0
2.36	0.37	12.32	0	0	13,425	10,264	0	0
2.37	0.39	14.63	0.5	0.25	13,590	10,389	164	124
2.38	0.29	13.75	0	0	13,425	10,264	0	0
2.39	0.53	10.74	0	0	13,425	10,264	0	0
2.40	0.23	10.74	0	0	13,425	10,264	0	0
2.41	0.39	21.94	0.5	0.5	13,590	10,524	164	260
2.42	0.38	20.29	0	0	13,425	10,264	0	0

Loss, all results are based on simulated or hypothetical scenarios.

## 5. Conclusions

The presented study investigates the impact of traffic sensor failure and/or detection accuracy on the toll computation by a dynamic toll pricing algorithm deployed on express lanes (ELs). The analysis methodology was demonstrated in a case study of tolled ELs on Interstate-95 in Florida, using the traffic and toll data for the Northbound and Southbound EL segments provided by FDOT for two selected dates in 2017. The existing dynamic toll pricing algorithm used for Interstate-95 ELs was studied and replicated analytically. The V-S relationships for ELs segments in the study area were developed and utilized to model erroneous sensor behavior. Two sets of experimental detection error scenarios (with several experiments in each) were designed and performed. A set of performance measures was used to investigate the impact of failed sensors and erroneous detection on EL tolls. The following conclusions have been reached:

- Uniformly applied detection errors (±5, 10, 15, and 20%) for all of the sensors on the Southbound segment show consistent results—underestimated traffic volumes result in a loss of the gross tolls. In contrast, the overestimated volumes result in a surplus of the tolls.
- Uniformly applied detection errors (±5, 10, 15, and 20%) for all of the sensors on the Northbound segment show that change in total gross toll occurs when the volume is overestimated by more than 15% for a day with an average traffic volume. In contrast, the detection error as low as 10% caused changes in toll amounts for a day with high traffic volumes.
- Considering the demonstrated cases in which detection errors of 5–10% did not trigger any changes in the tolls in the Northbound direction, the authorities could require that the accuracy of their detection systems cannot be lower than 90%.
- Experiments with multiple failed/erroneous sensors lead to results that are not easy to interpret systematically. In these experiments, the results vary between losses and gains based on the percentages of failed sensors and the magnitude of introduced errors on each sensor. The unpredictability of the resulting tolls appears to be more significant on the Southbound segment because it is more dynamic, traffic-wise, than the Northbound segment.
- In summary, the findings show that the detection errors greater than 10% should present a concern for the toll authorities in terms of possible underestimates or overestimates of reported traffic volumes, which reduce the algorithm’s effectiveness in determining the appropriate toll rates.

To further generalize the conclusions of this research, a future study is needed to analyze more Els segments in other geographic and operational conditions. Future studies should also focus on comparing various algorithms with different price sensitivities to the detected traffic conditions. The future research should also seek to develop an ‘index of dynamism’ of the EL, which would help determine how dynamic (or responsive) the dynamic tolls really are. Such information can then be used to update the day-of-week/time-of-day toll tables accordingly.

## Figures and Tables

**Table 1:** Variable notation.

Variable	Description
*TD*	Traffic link density (veh/mile/ln)
*F_TL_*	Traffic link flow (vehicles per hour)
*S_TL_*	Traffic link speed (miles per hour)
*NL*	Number of lanes
*V_TLC_*	Traffic link volume count (vehicles per data collection interval)
*DCI*	Data collection interval (900 s = 15 min)
*S_i_*	Average of one vehicle’s speeds (miles per hour)
*TD_c_*	Calculated traffic density (veh/mile/ln)
*TD_p_*	Previously calculated traffic density (veh/mile/ln)
Δ*TD*	Change in traffic density (veh/mile/ln)
Δ*TR*	Toll rate adjustment (USD)
*TR_s or p_*	Seed or previously calculated toll rate (USD)
*LOS_e_*	Existing level of service (A, B, C, D, E, or F)
*TR_i_*	Initial toll rate (USD)
*TR_f_*	Final toll rate (USD)

**Table 2:** Experiments of systematic detection error in Scenario 1.

Experiments ID’s of Subset 1 (Uniform Errors Applied for All Sensors)									
**Uniform Error**	0%	+5%	−5%	+10%	−10%	+15%	−15%	+20%	−20%
**Experiment ID—Southbound**	1.1	1.2	1.3	1.4	1.5	1.6	1.7	1.8	1.9
**Experiment ID—Northbound**	1.10	1.11	1.12	1.13	1.14	1.15	1.16	1.17	1.18
**Experiments ID’s and Used Sensor for Each Experiment in Subset 2**									
**Experiment ID**	**Southbound**				**Exp.ID**	**Northbound**			
	**Y% of Sensors Failed**		**Used Sensor(s)**			**Y% of Sensors Failed**		**Used Sensor(s)**	
1.19	25%		14.3,14.9,15.1		1.23	33.33%		14.5,14.9	
1.20	50%		14.6,15.1		1.24	66.67%		14.6	
1.21	75%		14.9						
1.22	100%		Time of Day table		1.25	100%		Time of Day table	

**Table 3:** Experiments of random detection error in Scenario 2.

Random Detection Error Scenarios for Southbound Segment				
Experiment ID	Y% of Sensors Failed	Used Sensor(s)	X% Modeled Error	Sensor(s) Subjected to X% Error, Respectively
2.1	0%	14.3,14.6,14.9,15.1	+5, +15%	14.3,14.6
2.2	0%	14.3,14.6,14.9,15.1	−5, −15%	14.3,14.6
2.3	0%	14.3,14.6,14.9,15.1	+10, +5%	14.6,14.9
2.4	0%	14.3,14.6,14.9,15.1	−10, −5%	14.6,14.9
2.5	0%	14.3,14.6,14.9,15.1	+10, +15, +5%	14.3,14.9,15.1
2.6	0%	14.3,14.6,14.9,15.1	−10, −15, −5%	14.3,14.9,15.1
2.7	0%	14.3,14.6,14.9,15.1	+10, +10, +20%	14.3,14.6,15.1
2.8	0%	14.3,14.6,14.9,15.1	−10, −10, −20%	14.3,14.6,15.1
2.9	0%	14.3,14.6,14.9,15.1	+15%	14.9
2.10	0%	14.3,14.6,14.9,15.1	−15%	14.9
2.11	25%	14.3,14.9,15.1	+10, +15%	14.9,15.1
2.12	25%	14.3,14.9,15.1	−10, −15%	14.9,15.1
2.13	25%	14.3,14.9,15.1	+10, +15, +20%	14.3,14.9,15.1
2.14	25%	14.3,14.9,15.1	−10, −15, −20%	14.3,14.9,15.1
2.15	50%	14.6,15.1	+15%	15.1
2.16	50%	14.6,15.1	−15%	15.1
2.17	50%	14.3,15.1	+10, +20%	14.3,15.1
2.18	50%	14.3,15.1	−10, −20%	14.3,15.1
2.19	75%	14.9	+10%	14.9
2.20	75%	14.9	−10%	14.9
2.21	75%	14.3	+15%	14.3
2.22	75%	14.3	−15%	14.3
**Random detection error scenarios for Northbound segment**				
2.23	0%	14.5,14.6,14.9	+10, +15, +20%	14.5,14.6,14.9
2.24	0%	14.5,14.6,14.9	−10, −15, −20%	14.5,14.6,14.9
2.25	0%	14.5,14.6,14.9	+10, +20%	14.5,14.6
2.26	0%	14.5,14.6,14.9	−10, −20%	14.5,14.6
2.27	0%	14.5,14.6,14.9	+10, +10%	14.5,14.9
2.28	0%	14.5,14.6,14.9	−10, −10%	14.5,14.9
2.29	0%	14.5,14.6,14.9	+5, +20%	14.6,14.9
2.30	0%	14.5,14.6,14.9	−5, −20%	14.6,14.9
2.31	33.33%	14.5,14.6	+15%	14.9
2.32	33.33%	14.5,14.6	−15%	14.9
2.33	33.33%	14.5,14.6	+10%	14.9,15.1
2.34	33.33%	14.5,14.6	−10%	14.9,15.1
2.35	33.33%	14.5,14.6	+10, +15%	14.3,14.9,15.1
2.36	33.33%	14.5,14.6	−10, −15%	14.3,14.9,15.1
2.37	33.33%	14.5,14.6	+15, +20%	15.1
2.38	33.33%	14.5,14.6	−15, −20%	15.1
2.39	66.67%	14.6	+10%	14.6
2.40	66.67%	14.6	−10%	14.6
2.41	66.67%	14.9	+20%	14.9
2.42	66.67%	14.9	−20%	14.9

## Data Availability

Data and codes used for this research could be shared with the research community on request to the corresponding author.
